# Single-molecule junction spontaneously restored by DNA zipper

**DOI:** 10.1038/s41467-021-25943-3

**Published:** 2021-10-01

**Authors:** Takanori Harashima, Shintaro Fujii, Yuki Jono, Tsuyoshi Terakawa, Noriyuki Kurita, Satoshi Kaneko, Manabu Kiguchi, Tomoaki Nishino

**Affiliations:** 1 Department of Chemistry, School of Science, Tokyo Institute of Technology, 2-12-1 W4-11 Ookayama, Meguro-ku, Tokyo 152-8551 Japan; 2 Department of Biophysics, Graduate School of Science, Kyoto University, Kitashirakawa-Oiwakecho, Sakyo, Kyoto 606-8502 Japan; 3 Department of Computer Science and Engineering, Toyohashi University of Technology, Tempaku-cho, Toyohashi, 441-8580 Japan

**Keywords:** DNA, Molecular electronics

## Abstract

The electrical properties of DNA have been extensively investigated within the field of molecular electronics. Previous studies on this topic primarily focused on the transport phenomena in the static structure at thermodynamic equilibria. Consequently, the properties of higher-order structures of DNA and their structural changes associated with the design of single-molecule electronic devices have not been fully studied so far. This stems from the limitation that only extremely short DNA is available for electrical measurements, since the single-molecule conductance decreases sharply with the increase in the molecular length. Here, we report a DNA zipper configuration to form a single-molecule junction. The duplex is accommodated in a nanogap between metal electrodes in a configuration where the duplex is perpendicular to the nanogap axis. Electrical measurements reveal that the single-molecule junction of the 90-mer DNA zipper exhibits high conductance due to the delocalized π system. Moreover, we find an attractive self-restoring capability that the single-molecule junction can be repeatedly formed without full structural breakdown even after electrical failure. The DNA zipping strategy presented here provides a basis for novel designs of single-molecule junctions.

## Abstract (web summary)

The versatility of DNA has inspired many single-molecule investigations utilizing nanotechnology. Harashima et al. have a somewhat different take on the subject and study a zipper configuration bridging electrodes that resembles an active electro-mechanical component instead.

## Introduction

The structural, physical, and chemical properties of DNA at the nanoscale have attracted attention to the prospect of using DNA as a building block in nanoscience. In the field of molecular electronics, electron transport through a single DNA molecule has been extensively investigated^1–5^. These studies exploit single-molecule junctions, in which a DNA molecule bridges a nanogap between the metal electrodes. For example, it was reported that A-form and B-form DNAs differ in the single-molecule conductance by an order of magnitude^6^. Many studies have revealed that there is a change in the electron transport properties of DNA after ligand binding^7–9^. These include an intercalator whose perturbation of base stacking leads to switching or rectifying properties of DNA^10–12^. Moreover, a three-terminal DNA connected by a guanine quadruplex has been exploited to construct a single-molecule junction, and it was demonstrated that the intricate junction structure shows excellent functionality for a charge splitter^13^. Significant advances have been made in understanding the transport phenomena through a single DNA molecule, as exemplified above. However, these previous studies mainly focused on the transport phenomena in static DNA structure at thermodynamic equilibria.

Recently, rational control of DNA structures at the single-molecule level has been achieved by modern techniques and emerging technologies. Capturing desired conformation and separation of DNA are achieved by elaborate flow in nanofluidic systems^14–17^. Sequencing analysis of DNA at the single-base resolution can be performed with nanopore devices during the constrained passage^18–21^. Also, precise control of DNA structures by atomic force microscopy (AFM) has enabled accurate mapping of DNA unzipping dynamics and a free-energy landscape^22–24^. This combination of electrical measurements and structural modulation of DNA could lead to the realization of the sophisticated functionality of electronic devices based on a single DNA molecule. For example, we expect that electron transport through DNA under the deliberate control of its structure paves the way for DNA electronic devices with functional controllability in a dynamic manner.

**Fig. 1:**
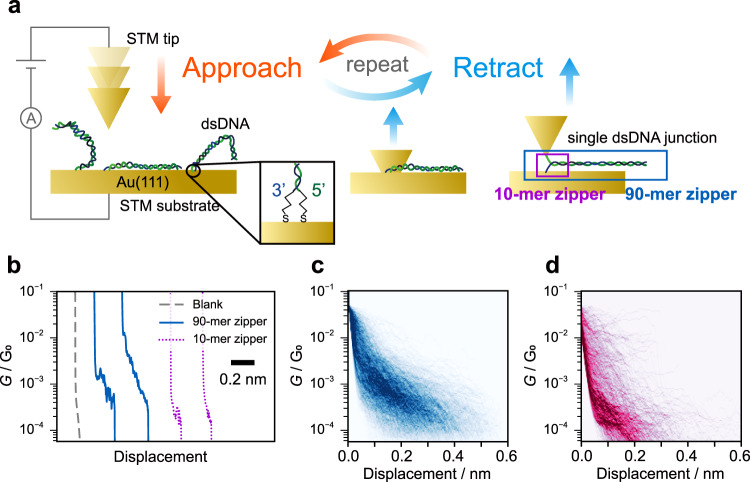
Single-molecule junction of DNA zipper. **a** Schematic illustration of the scanning tunnelling microscopy–break-junction (STM-BJ) measurements. **b** Representative conductance traces for unmodified Au(111) substrate (graydashed lines), and 90-mer and 10-mer DNAs (blue solid lines and purple dotted lines, respectively). **c** and **d** 2D histograms of the conductance–displacement (*G–z*) traces for 90-mer and 10-mer DNAs, respectively. The origin of the displacement was set at the point where the conductance decreased below 50 mG_0_. 2219 and 2635 traces were analyzed for histograms in **c** and **d**, respectively. Tip velocity, 31 nm/s; bias voltage, 20 mV.

Here, we report the investigation of electron transport through the single-molecule junction of a zipper DNA that orthogonally clamps a metal nanogap (see Fig. 1a). The present DNA single-molecule junction differs from conventional ones in the DNA configuration; the present and conventional junctions contain a DNA molecule oriented in perpendicular and parallel directions to the axis of the nanogap, respectively. The DNA single-molecule junction with the present zipper configuration exhibited high single-molecule conductance that cannot be achieved using DNA junctions with the conventional configuration. The unzipping dynamics of the molecular junction were characterized by tunnelling currents in break-junction (BJ) experiments, based on scanning tunnelling microscopy (STM). The STM measurement and the molecular dynamics (MD) simulations of the unzipping dynamics revealed that the DNA junction with zipper configuration enables spontaneous restoration of the molecular junction after its electrical failure and thereby improves the reproducibility of the junction formation. Our study demonstrated that a DNA dynamic structural change could be applied to a single-molecule junction by using the zipper configuration. The findings pave way for novel functionality and superior properties of nanoscale electronic devices^22,25^.

## Results and discussion

### Single-molecule conductance

First, STM-BJ experiments were performed to measure conductance of the single-molecule junction of DNA with a zipper configuration. Either 90-mer or 10-mer DNA was employed as a sample molecule. One strand of the sample was functionalized at the 3′ end with a thiol linker, while the same linker was introduced at the 5′ end of another strand (Fig. 1a). These ends were tethered to the STM tip or Au(111) surface. The STM tip was repeatedly brought in and out of contact with the Au(111) surface modified with the DNA duplex. The conductance was monitored as a function of the tip displacement during the retraction process (Fig. 1b). The resulting two-dimensional (2D) histogram, where thousands of conductance–displacement (*G*–*z*) traces were overlaid, clearly shows conductance plateaus at 1.9 and 0.15 mG_0_ for 90-mer and 10-mer DNA, respectively (Fig. 1c, d). The difference in the single-molecule conductance is discussed later. The single plateau and subsequent conductance decay in the traces indicate that these plateaus are attributed to the single-molecule junction that contains DNA. The tunnelling decay constants during and after the plateau (β_1_ and β_2_, respectively) were analysed from each conductance trace, and β_1_ and β_2_ for 90-mer DNA were determined to be 0.27 and 2.0 Å^−1^, respectively (see Supplementary Note 1 for the results of 10-mer DNA and detailed discussion of STM-BJ results). The decay constants are known to depend on the energy gap between the Fermi level of the electrode and that of the molecular orbital for the tunnelling transport^26^. The β_2_ value found here is consistent with that for direct tunnelling between the tip and substrate without the molecular junction (2.2 Å^−1^, Supplementary Note 1). On the other hand, the β_1_ value is smaller than the typical value for alkanedithiol, but similar to the ones for π-conjugated molecules^26^, which indicates that the electron transport involves the DNA. The DNA zipper junction transmits electrons in the transverse direction, and it is anticipated that the base pairs, especially those located at the DNA terminal, mediate the electron transport (see Fig. 1a). In this case, the transport properties of the present junction can be compared with those of the single-molecule junction of DNA bases, which have been investigated toward the realization of single-molecule sequencing^27–30^. Indeed, the reduction in the decay constants as observed for β_1_ value in the present experiments was reported for the tunnelling through the DNA base pairs^31,32^. Further STM-BJ experiments showed that the conductance of the molecular junction reflects the DNA sequence (Supplementary Note 2), which further supports that the electron transport is mediated by the DNA.

The present DNA single-molecule junction possesses a different configuration from the conventional one. In the present study, the DNA duplex bears the two linker groups at the same end. Thus, the molecular junction contains DNA in a configuration orthogonal to the axis of the gap between the tip and substrate (Fig. 1a). In contrast, the linker groups were conventionally located at the opposite ends of the duplex, and the junction accommodates the duplex aligned parallel to the gap axis. Electron transport through the latter conventional junction steeply attenuates with increased DNA length, since the electrons travel through the whole duplex^33^. No such attenuation happens in the present single-molecule junction. Indeed, the conductance of the 90-mer DNA zipper junction is larger than that of the 10-mer zipper junction. We measured the conductance of the zipper junction of a variety of DNA lengths ranging from 10 to 90 base pairs and confirmed that the conductance value increased as the DNA length increased (Supplementary Note 3). This length dependence, together with the self-restoring capability described later, are the advantages of the present configuration.

### Transition voltage spectroscopy

**Fig. 2:**
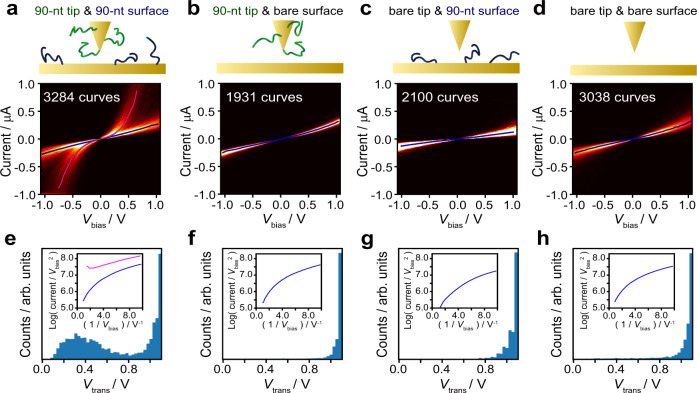
Current–voltage (*I–V*) and transition voltage spectra. *I–V* 2D histograms and *V*_trans_ histograms obtained with (**a, e**) molecular tip and modified substrate, (**b, f**) molecular tip and bare substrate, (**c, g**) unmodified tip and modified substrate, and (**d, h**) unmodified tip and bare substrate. Bias voltage (*V*_bias_) was swept from −1.1 V to 1.1 V in 5 ms. Representative transition voltage spectra are shown in insets of **e**–**h**. Pink and blue spectra correspond to those with low and high *V*_trans_, respectively.

Next, current–voltage (*I–V*) curves of the single-molecule DNA junction with the zipper configuration were obtained to investigate the electron transport properties in detail. We previously developed a methodology to study electron transport induced by in situ hybridization of a single DNA duplex using an STM molecular tip^7,34^. This technique was also utilized in the present study: an Au STM tip was modified with a single strand of the 90-mer DNA zipper. An Au(111) substrate was separately modified with single-stranded DNA (ssDNA) complementary to the strand on the tip (Fig. 2a). After the molecular tip was brought near to, but never in contact with, the surface, the bias voltage was swept with the tip as the sample distance was held stationary to acquire the *I–V* curves. Figure 2a shows the resulting *I*–*V* curves, and Fig. 2b–d presents those obtained in the control experiments, i.e., the measurements with the molecular tip and unmodified surface, with the unmodified tip and ssDNA-modified substrate, and with the unmodified tip and unmodified surface, respectively. The *I–V* curves in Fig. 2a clearly exhibited two distributions, i.e., states with high and low conductance. The low-conductance state was common to those found in the control experiments (Fig. 2b–d), indicating that this state stems from the gap devoid of the DNA bridge. The high-conductance state was thus ascribed to the molecular junction of the DNA zipper. The conductance as estimated by the *I–V* properties agrees with the conductance as determined by the BJ studies (Fig. 1c, d, see Supplementary Note 4), supporting the assignment of a high-conductance state to the DNA junction. It has been reported that ssDNA can adsorb to a metal surface via its bases^35^. However, no state that could be attributed to ssDNA molecular junctions was found in the *I–V* curves in Fig. 2b, c. STM-BJ study was also conducted with the unmodified tip and the ssDNA-modified substrate, and the conductance histograms without notable peaks were obtained (Supplementary Note 5). These results are most probably due to the significantly decreased conductance of ssDNA as compared to that of dsDNA because of base stacking is less ordered in ssDNA^36^. The conductance of the ssDNA junction would be almost indistinguishable from the conductance of the gap without the dsDNA bridge in the present measurements.

For evaluation of the electronic structure of the molecular junction, the transition voltage (*V*_trans_) was estimated using *I–V* curves of the high-conductance state (Fig. 2e–h). It has been known that *V*_trans_ is proportional to the energy gap between the Fermi level of the electrode (the tip or the substrate) and that of the conduction orbital of the molecule in the junction^37–39^. In transition voltage spectra, log(*I*/*V*^2^) is plotted against *V*^–1^, and the voltage at which the plot reaches a minimum corresponds to *V*_trans_^37–39^. The mean value of *V*_trans_ for the molecular junction of the 90-mer DNA zipper was found to be 0.4 V (Fig. 2e). In our previous work, a *V*_trans_ value of 0.8 V was found for a single-molecule junction of 8-mer DNA in the conventional configuration. It was further observed that this value decreased to 0.5 V upon binding of an intercalator to the DNA^11^. The *V*_trans_ value for the molecular junction of the DNA zipper is smaller than in both these above-mentioned cases. This result suggests the decrease of the energy gap by delocalization of π stacking orbitals over its long base pairs, which agrees with previous research that used fragment molecular orbital and density-functional theory calculations^40,41^. We attribute the small *V*_trans_, that is, the decreased energy gap, to delocalization of the π-orbitals of DNA over its long base pairs. To prove this, molecular orbital calculations were performed based on density-functional theory (Supplementary Note 6). We indeed found that the energy gap between the highest occupied molecular orbital (HOMO) and the lowest unoccupied molecular orbital (LUMO) decreased with the increase in DNA length, in line with previous theoretical studies^40,41^. The larger conductance of the 90-mer DNA junction compared to the 10-mer counterpart (Fig. 1c) is consistent with the length-dependent decrease in the HOMO–﻿LUMO gap. Thus, we conclude that the single-molecule junction of the DNA zipper attains high conductance due to the delocalized π system of the stacked DNA bases near the electrodes. The effect of this electron delocalization could explain the higher conductance of the 90-mer DNA zipper compared with that of the 10-mer counterpart (Fig. 1b), though this behavior merits further investigation.

### Self-restoring capability

**Fig. 3:**
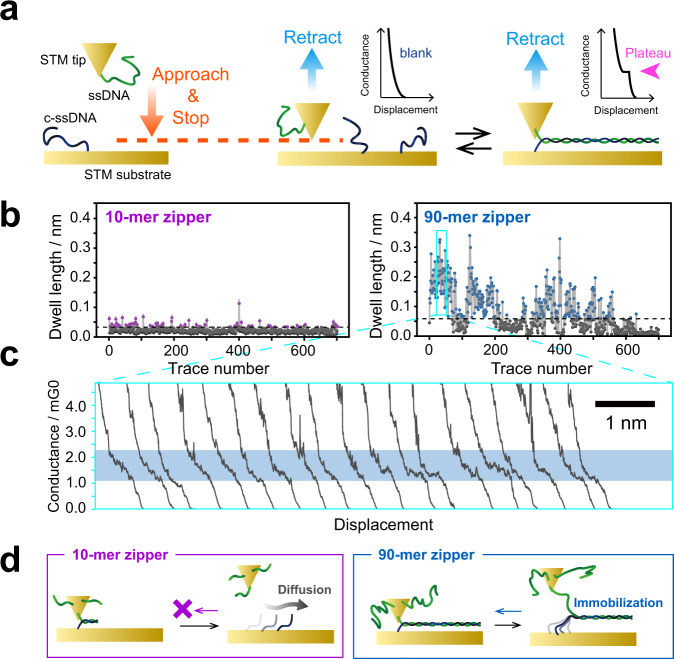
Consecutive conductance–displacement (*G*–*z*) measurements for molecular junctions of DNA zipper. **a** Schematic illustration of in-situ hybridization using STM molecular tip. Initial set-point current, 7.5 nA; pull-up distance, 30 nm; tip velocity, 31 nm/s. **b** Temporal evolution of the dwell length of *G*–*z* trace for 10-mer (left) and 90-mer (right) DNA. The dwell length was calculated as the trace length between 0.14 and 0.16 mG_0_ and between 1.4 and 2.4 mG_0_ for the 10-mer and 90-mer zipper, respectively. The threshold values for 10-mer and 90-mer DNA were 0.028 and 0.060 nm, respectively, as indicated by dashed lines. **c** Successive *G–z* traces, extracted from the area indicated by square in **b**. **d** Plausible model for the formation and breakdown of molecular junctions in DNA zipper.

Highly feasible and reproducible formation of a single-molecule junction, in addition to high conductivity, is a critical step toward the realization of electronic devices using a single DNA molecule. The zipping configuration of the present molecular junction enabled us to employ a longer DNA duplex, which is known to improve the thermodynamic stability of the duplex in solution. Thus, we expect that the present strategy could improve the stability and/or the reproducibility of single-molecule junctions. To test this hypothesis, we investigated repeated formation of the single-molecule junction of the DNA zipper (Fig. 3a). An ssDNA molecule and its complementary strand were tethered to the STM tip and the Au(111) surface, similar to that in the *I*–*V* measurement. The molecular tip was first carefully brought in close proximity to the sample surface under the STM feedback loop. After awaiting for 0.3 s to facilitate hybridization and formation of the zipper structure, the STM tip was pulled up by 30 nm to record the conductance trace. The whole process was repeated to record consecutive *G*–*z* traces. For each trace, the dwell length was determined as the trace length between 1.4 and 2.4 mG_0_ and between 0.14 and 0.16 mG_0_ for the 90-mer and 10-mer zipper, respectively. These ranges were determined on the basis of the conductance values and their standard deviations from the STM-BJ measurements (see Fig. 1b). Figure 3b shows the time course of the dwell length obtained by successive 700 *G*–*z* traces for 10-mer and 90-mer DNA. The dwell length was then compared to the plateau length of the single-molecule junctions (dashed lines in Fig. 3b) of the 90-mer or 10-mer zipper DNA (Supplementary Note 1) to determine whether the molecular junction was successfully formed. It is clear that the 90-mer DNA zipper structure significantly enhances the repeatability of formation of the single-molecule junction compared with that of the 10-mer DNA zipper structure. The maximum number of repeated junction formations of the 90-mer DNA zipper reached 78; the single-molecule junction of the 90-mer DNA zipper was repeatedly reproduced for approximately 100 s despite the repeated perturbation of the junction by the tip displacements (Fig. 3c). The time course of the dwell length was quantitatively analyzed using joint probabilities (Supplementary Note 7). The analysis led to the same conclusion: the repeated and random formation of the molecular junction for 90-mer and 10-mer DNAs, respectively. The repeated formation of the DNA zipper junction was also confirmed by measuring mechanical forces exerted on the junction with AFM (Supplementary Note 8). A plausible model of DNA dynamics in this experiment was proposed in Fig. 3d. Repeated formation of the molecular junction for the 90-mer DNA could be due to partial preservation of the DNA duplex during pull-up procedures in the current measurements. This is not the case for the single-molecule junction of the 10-mer DNA zipper, since the pull-up distance of 30 nm is enough to break this junction considering the length of the duplex. The displacement dependence of the restoration behavior demonstrates the participation of the partially hybridized duplex and thus corroborates this model (Supplementary Note 9). The self-restoring behavior found for the present zipper junction opens up a way for reliable operations of single-molecule devices.

### MD simulations

**Fig. 4:**
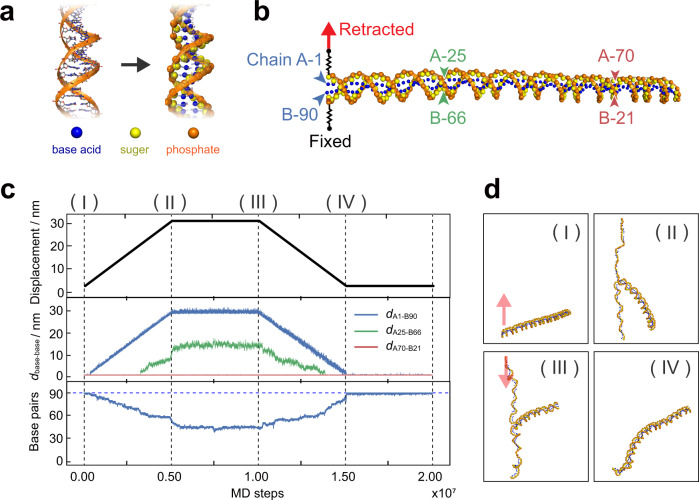
Course-grained molecular dynamics (MD) simulations. **a** Schematic illustration of DNA modelling. Nucleotides are represented by three spherical sites corresponding to the nitrogenous base (blue), deoxyribose sugar (yellow), and phosphate group (orange). **b** Model of single-molecule junction of 90-mer DNA. Arrowheads indicate the selected bases. **c** Time course of displacement of moving end (top), the distance between complementary bases (middle), and the number of base pairs in the duplex region (bottom). **d** Snapshots of simulated structures of the zipper DNA. Labels (I)–(IV) correspond to MD steps shown in **c**.

Course-grained MD simulations were performed to confirm partial preservation of the duplex for the single-molecule junction in the DNA zipper configuration. The CafeMol software^42^ with 3SPN.2 C model^43,44^ was used for these calculations. It has been shown that this model successfully reproduces melting phenomena, persistence lengths, and major and minor grooves of dsDNA^43–45^. In the present work, one end of the 90-mer DNA duplex in the single-molecule junction was linked to two springs to represent the STM experiments (Fig. 4a, b). Then, the terminal base in one strand of the duplex (Chain A in Fig. 4b) was gradually pulled up by 30 nm in the simulation, as in the experiments (Fig. 3d). This base was then moved down to the initial position after the waiting time, as shown in the top panel of Fig. 4c. The distances between the selected complementary bases and the number of base pairs contained in the duplex region were plotted in the middle and bottom panels of Fig. 4c, respectively. Figure 4d shows selected snapshots of the single-molecule junction. Crucially, the DNA duplex is partially preserved after retraction (Fig. 4c, (II)), and the partial duplex, containing 44 base pairs, persisted after the waiting time (Fig. 4c, (III)). As the tethered end was pushed back to its original position after retraction, the unwound portion of DNA gradually reannealed to restore the complete duplex (Fig. 4c, (IV); see also Supplementary Movie 1). The distances between the complementary bases remained unchanged at the DNA end opposite to the tethered base (Fig. 4c, middle), demonstrating again the partial preservation of the DNA duplex. We carried out additional simulations, where the tethered end was released after the pull-up of 30 nm. The reannealing also occurred in this case, as found for the simulations above, and the complete duplex emerged (Supplementary Movie 2). These findings prove that spontaneous base-pairing accounts for duplex restoration, and not the external force exerted by the spring, which represents the STM tip in the experiments.

The lifetime of conventional single-molecule junctions, such as those of alkanedithiol, is typically short (in the order of milliseconds at room temperature), which poses fundamental problems for the realization of single-molecule electronics. These junctions break at the interface between the constituent molecule and the electrode, because the bonding between the functional group of the molecule and the electrode, e.g., Au–﻿S bond, is weakest in the junction^46^. It has been reported that the force required to break the Au–﻿S or Au–﻿Au bond is 1–2 nN^47^, while the DNA unzipping takes place under the force of 10–50 pN^22–24^. Thus, in the present junctions, the breakdown is attributed to be via DNA unzipping. The unzipping relaxes the tensile stress on the junction. Therefore, the duplex of long DNA zippers can be partially preserved, leading to prolonged preservation of the single-molecule connection with a DNA zipper. These results demonstrate that the DNA zipper offers a means to achieve both reproducible formation by the self-restoring capability and high conductivity.

In summary, we developed a DNA single-molecule junction where the duplex orthogonally clamped the metal nanogap. The electrical measurements based on STM revealed that the molecular junction of the DNA zipper attains high conductance, originating from the delocalized π system of the DNA near the electrodes. Moreover, we found an attractive self-restoring capability that the single-molecule junction can be repeatedly formed without breakdown of the whole structure even after an electrical failure. This advantage arises from the partial unzip of the DNA within the junction, which is supported by the coarse-grained MD simulations. It is also worth noting that conductance of the molecular junction exhibited no steep dependence on DNA length, which is distinctly different from the conventional single-molecule junction of DNA. Therefore, we expect that further functionalization could be possible through the use of a long DNA sequence as a scaffold to form conjugates with a wide range of functional (bio)molecules.

## Methods

### Sample preparation

An Au(111) surface was prepared by thermal evaporation on a mica surface. Au wires (99.999%, 0.25 mm diameter) were chemically etched with 3.0 M NaCl solution in 1% perchloric acid (HClO_4_) to prepare the STM tips. We used 90-mer DNA (Supplementary Table 1) modified with 1,3-propanethiol [–﻿(CH_2_)_3_SH] linkers at the hydroxy group of the 3′ end as the probe. The complementary strand was modified with –﻿(CH_2_)_3_SH linkers at the phosphate group of the 5′ end. As a control, we employed 10-mer DNA (Supplementary Table 1), which is partially the same as the 90-mer DNA, and –﻿(CH_2_)_3_SH linkers were introduced at the 3′ end. The complement strand was modified with –﻿(CH_2_)_3_SH linkers at the 5′ end. All the DNAs, synthesized by solid-phase synthesis using the phosphoramidite method^48^, purified by high-performance liquid chromatography, and characterized by time-of-flight mass spectrometry (Supplementary Note 10), were purchased from Tsukuba Oligo Service (Ibaraki, Japan). For the preparation of the duplex, 1 μM solutions of ssDNAs were mixed with 10 mM phosphate-buffered saline (PBS) solution. This mixture was heated at 75 °C for 45 min. and slowly cooled to room temperature. The Au substrate was immersed in the solution for at least 2 h. For the experiments involving the molecular tip, STM tips were immersed in 1 μM ssDNA in PBS solution.

### STM measurement

STM measurements were performed on an SPM 5100 system (Agilent Technologies, Santa Clara, CA, USA). The tunnelling current was sampled at 20,000 Hz. The bias voltage was 20 mV in all experiments, except for the *I–V* measurement. In the analyses, conductance traces with a simple exponential decay were removed based on an automated algorithm, in which the presence or absence of a plateau was judged using the conductance histogram constructed for each conductance trace^49^. Current measurements were repeated using independently prepared tips and sample surfaces. The reproducibility was confirmed by comparing conductance histograms obtained using every independent sample surface.

### Theoretical calculation

We conducted the course-grained MD simulation with CafeMol^42^ software. The 3SPN.2C model developed by the de Pablo group was used for DNA^44^. All the simulations were performed at 300 K. The DNA sequence as employed in the experiments was used for the simulation. For simulating the experiment in ambient conditions, no electrostatic interactions were taken into consideration. One terminus in each DNA strand was connected with springs for modelling the bridge between thiol linkers of DNA and Au atoms. The stiffness of the spring was 8.5 N/m, which is equal to the strength of the Au–﻿Au bond^47,50^.

### Reporting Summary

Further information on research design is available in the Nature Research Reporting Summary linked to this article.

## Supplementary information

Supplementary Information

Peer Review File

Description of Additional Supplementary Files

Supplementary Movie 1

Supplementary Movie 2

Reporting Summary

## Data Availability

The data that support the findings of this study are available from the corresponding author upon reasonable request. Source data for Figs. 1–4 and Supplementary Fig. 1–12 are archived at 10.5281/zenodo.5515109. Source data are provided with this paper.

## Source data

Source Data

## References

[1] Kelley SO, Barton JK (1999). Electron transfer between bases in double helical DNA. Science.

[2] Risser SM, Beratan DN, Meade TJ (1993). Electron-transfer in DNA - predictions of exponential-growth and decay of coupling with donor-acceptor distance. J. Am. Chem. Soc..

[3] Tsutsui M (2011). Electrical detection of single methylcytosines in a DNA oligomer. J. Am. Chem. Soc..

[4] Xiang LM (2015). Intermediate tunnelling-hopping regime in DNA charge transport. Nat. Chem..

[5] Xu BQ, Zhang PM, Li XL, Tao NJ (2004). Direct conductance measurement of single DNA molecules in aqueous solution. Nano Lett..

[6] Artes JM, Li YH, Qi JQ, Anantram MP, Hihath J (2015). Conformational gating of DNA conductance. Nat. Commun..

[7] Bui PT, Nishino T, Shiigi H, Nagaoka T (2015). One-by-one single-molecule detection of mutated nucleobases by monitoring tunneling current using a DNA tip. Chem. Commun..

[8] Lewis FD (2000). Direct measurement of hole transport dynamics in DNA. Nature.

[9] Wierzbinski E (2013). The single-molecule conductance and electrochemical electron-transfer rate are related by a power law. Acs Nano.

[10] Guo CL (2016). Molecular rectifier composed of DNA with high rectification ratio enabled by intercalation. Nat. Chem..

[11] Harashima T, Kojima C, Fujii S, Kiguchi M, Nishino T (2017). Single-molecule conductance of DNA gated and ungated by DNA-binding molecules. Chem. Commun..

[12] Xiang LM (2017). Gate-controlled conductance switching in DNA. Nat. Commun..

[13] Sha R (2018). Charge splitters and charge transport junctions based on guanine quadruplexes. Nat. Nanotechnol..

[14] Azad Z, Roushan M, Riehn R (2015). DNA brushing shoulders: targeted looping and scanning of large DNA strands. Nano Lett..

[15] Jadhav V, Hoogerheide DP, Korlach J, Wanunu M (2019). Porous zero-mode waveguides for picogram-level DNA capture. Nano Lett..

[16] Riehn R, Austin RH, Sturm JC (2006). A nanofluidic railroad switch for DNA. Nano Lett..

[17] Terakawa T (2017). The condensin complex is a mechanochemical motor that translocates along DNA. Science.

[18] Raillon C (2012). Nanopore detection of single molecule RNAP-DNA transcription complex. Nano Lett..

[19] Raillon C, Granjon P, Graf M, Steinbock LJ, Radenovic A (2012). Fast and automatic processing of multi-level events in nanopore translocation experiments. Nanoscale.

[20] Heerema SJ, Dekker C (2016). Graphene nanodevices for DNA sequencing. Nat. Nanotechnol..

[21] Xie P, Xiong QH, Fang Y, Qing Q, Lieber CM (2012). Local electrical potential detection of DNA by nanowire-nanopore sensors. Nat. Nanotechnol..

[22] Krautbauer R, Rief M, Gaub HE (2003). Unzipping DNA oligomers. Nano Lett..

[23] Strunz T, Oroszlan K, Schafer R, Guntherodt HJ (1999). Dynamic force spectroscopy of single DNA molecules. Proc. Natl Acad. Sci. USA.

[24] Walder R (2018). High-precision single-molecule characterization of the folding of an HIV RNA hairpin by atomic force microscopy. Nano Lett..

[25] Cocco S, Monasson R, Marko JF (2001). Force and kinetic barriers to unzipping of the DNA double helix. Proc. Natl Acad. Sci. USA.

[26] Kiguchi M, Kaneko S (2013). Single molecule bridging between metal electrodes. Phys. Chem. Chem. Phys..

[27] Afsari S (2017). Quantum point contact single-nucleotide conductance for DNA and RNA sequence identification. Acs Nano.

[28] Huang S (2010). Recognition tunneling measurement of the conductance of DNA bases embedded in self-assembled monolayers. J. Phys. Chem. C..

[29] Huang S (2010). Identifying single bases in a DNA oligomer with electron tunnelling. Nat. Nanotechnol..

[30] Ohshiro T, Tsutsui M, Yokota K, Taniguchi M (2018). Quantitative analysis of DNA with single-molecule sequencing. Sci. Rep..

[31] Chang S (2009). Tunnelling readout of hydrogen-bonding-based recognition. Nat. Nanotechnol..

[32] Huang S (2010). Recognition tunneling measurement of the conductance of DNA bases embedded in self-assembled monolayers. J. Phys. Chem. C..

[33] Xu Zhang, Li Tao (2004). Direct conductance measurement of single DNA molecules in aqueous solution. Nano Lett..

[34] Nishino T, Bui PT (2013). Direct electrical single-molecule detection of DNA through electron transfer induced by hybridization. Chem. Commun..

[35] Kimura-Suda H, Petrovykh DY, Tarlov MJ, Whitman LJ (2003). Base-dependent competitive adsorption of single-stranded DNA on gold. J. Am. Chem. Soc..

[36] Cohen H, Nogues C, Naaman R, Porath D (2005). Direct measurement of electrical transport through single DNA molecules of complex sequence. Proc. Natl Acad. Sci. USA.

[37] Beebe JM, Kim B, Gadzuk JW, Frisbie CD, Kushmerick JG (2006). Transition from direct tunneling to field emission in metal-molecule-metal junctions. Phys. Rev. Lett..

[38] Guo S, Hihath J, Díez-Pérez I, Tao N (2011). Measurement and statistical analysis of single-molecule current–voltage characteristics, transition voltage spectroscopy, and tunneling barrier height. J. Am. Chem. Soc..

[39] Huisman EH, Guédon CM, van Wees BJ, van der Molen SJ (2009). Interpretation of transition voltage spectroscopy. Nano Lett..

[40] Kubar T, Elstner M (2013). A hybrid approach to simulation of electron transfer in complex molecular systems. J. R. Soc. Interface.

[41] Kashimoto Y (2018). The evolution of intermolecular energy bands of occupied and unoccupied molecular states in organic thin films. J. Phys. Chem. C..

[42] Kenzaki H (2011). CafeMol: a coarse-grained biomolecular simulator for simulating proteins at work. J. Chem. Theory Comput..

[43] Freeman GS, Lequieu JP, Hinckley DM, Whitmer JK, de Pablo JJ (2014). DNA shape dominates Sequence affinity in nucleosome formation. Phys. Rev. Lett..

[44] Hinckley DM, Lequieu JP, de Pablo JJ (2014). Coarse-grained modeling of DNA oligomer hybridization: Length, sequence, and salt effects. J. Chem. Phys..

[45] Terakawa T, Takada S (2015). p53 dynamics upon response element recognition explored by molecular simulations. Sci. Rep..

[46] Xu BQ, Xiao XY, Tao NJ (2003). Measurements of single-molecule electromechanical properties. J. Am. Chem. Soc..

[47] Rubio-Bollinger G, Bahn SR, Agrait N, Jacobsen KW, Vieira S (2001). Mechanical properties and formation mechanisms of a wire of single gold atoms. Phys. Rev. Lett..

[48] Beaucage SL, Iyer RP (1993). The functionalization of oligonucleotides via phosphoramidite derivatives. Tetrahedron.

[49] Inkpen MS (2015). New insights into single-molecule junctions using a robust, unsupervised approach to data collection and analysis. J. Am. Chem. Soc..

[50] Aradhya SV, Frei M, Hybertsen MS, Venkataraman L (2012). Van der Waals interactions at metal/organic interfaces at the single-molecule level. Nat. Mater..

