# Supplementary material for: Single-molecule junction spontaneously restored by DNA zipper
Source: Nat Commun. 2021 Oct 1;12:5762. doi: 10.1038/s41467-021-25943-3 (PMC8486845; doi:10.1038/s41467-021-25943-3)
Supplement: Supplementary file 1 — Supplementary Information [file 41467_2021_25943_MOESM1_ESM.pdf]

Supporting information:

Single-molecule junction spontaneously restored by DNA zipper

Authors: Takanori Harashima *et al*.

Supplementary Note 1: Tunneling decay constant and plateau length.

The conductance traces for the single-molecule junctions of the 10-mer and 90-mer DNA zippers were fitted to exponential functions according to the relation $G \propto \exp(-\beta z)$, where z and β are the gap width and the tunneling decay constant, respectively. Two exponential functions were used in the present study for the plateau and consecutive rapid decay regions, and consequently two decay constants were obtained, i.e., β_1 and β_2 for the plateau and the decay regions, respectively

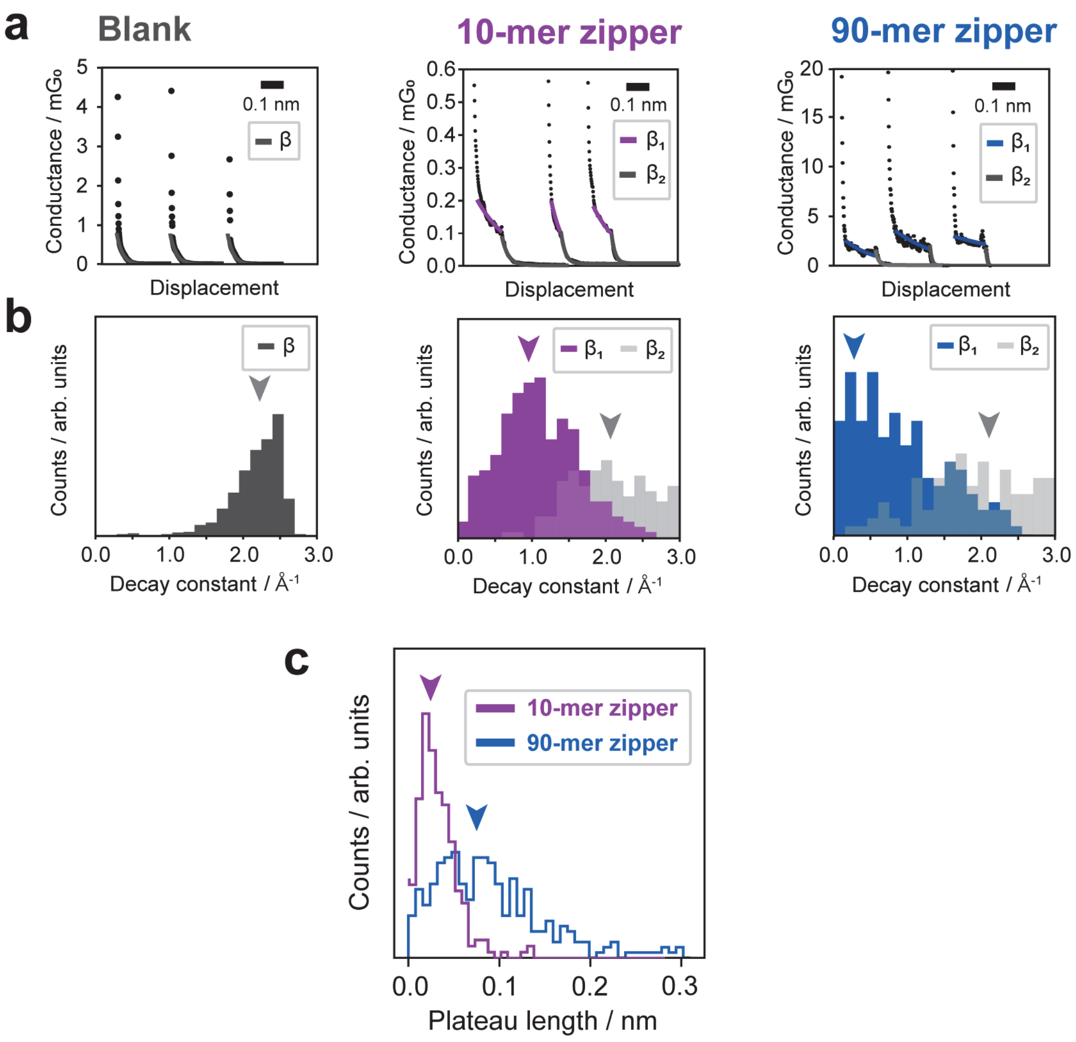

Supplementary Fig. 1. a Analysis of decay constants for the blank (left), the 10-mer (middle), and the 90-mer (right) DNA zippers. **b** Histograms of decay constants for the blank (left), the 10-mer (middle), and the 90-mer (right) DNA zippers. Arrowheads indicate the mean value of each histogram. **c** Plateau length histograms of 10-mer (purple solid line) and 90-mer (blue solid line) DNA. Arrowheads indicate the median values of the plateau length. 229 and 320 traces were used for 10-mer and 90-mer DNA, respectively.

(Supplementary Fig. 1a). The β_1 values were found to be 0.97 and 0.27 Å^{-1} for the 10-mer and 90-mer DNA junctions, respectively (Supplementary Fig. 1b). The smaller value of β_1 of the 90-mer DNA suggested a narrower energy difference between the Fermi level and molecular orbital than 10-mer DNA. The β_2 value obtained for 10-mer and 90-mer DNA were 2.1 and 2.0 Å^{-1}. In this case, the resulting values was constant, irrespective of the DNA used in the measurements. In addition, these values were consistent with the theoretical value of the vacuum gap between Au electrodes,[1] as expected for the decay regions after the rupture of the molecular junctions.

The fitting for β_1 allowed us to locate the plateau region and evaluate its length, which is equivalent to the distance from which the junction can be stretched before the breakdown, in each conductance trace. Supplementary Fig. 1c shows the histograms for the length of the resulting plateaus, and the median values of the plateau length for 10-mer and 90-mer DNA were determined to be 0.03 and 0.08 nm, respectively. Similar values of the plateau length were reported for the single-molecule junction of double-stranded DNA (dsDNA) in the conventional junction configuration, where the duplex aligned parallel to the axis of the gap between the STM tip and substrate[2]. The plateau length significantly shorter than the molecular length of the DNA has been ascribed to the shear force localized to the terminal base pairs. The mechanical stretch exerted on the DNA by the tip is not evenly distributed along the duplex but is localized at the end base pairs[3,4]. Consequently, even at the short plateau length, the large shear force arises to mechanically melt the DNA duplex at the termini. The similar plateau length detected in the present and previous works, though different junction configurations were employed, indicates that the breakdown of the present molecular junction that leads to the abrupt decrease in the conductance (see Supplementary Fig. 1a) is also caused by the dissociation of terminal base pair of the duplex. The mechanical stretch in the present experiments was applied directly to the DNA terminal in the direction to rupture the hydrogen bonding in the base pair, which rationalizes the aforementioned mechanism.

Supplementary Note 2: Effect of base sequence on conductance of DNA zipper junction.

We investigated the effect of the DNA sequence on the conductance of the single-molecule junction with the zipper configuration. A portion of the sequence of the sample 90-mer DNA was changed: the 30 bases located at the thiolated terminal were replaced with the base sequence of $(A_5T_5)_3$. STM-BJ measurements were carried out using an Au(111) substrate modified with this "mutated" DNA. The *G*–*z* traces exhibited plateaus (Supplementary Fig. 2a), demonstrating the formation of the DNA zipper junction. The statistically most probable conductance of the junction was found to be 0.13 mG_0, based on the histogram analysis of the traces (Supplementary Fig. 2b). Notably, this value was smaller than the conductance for the junction of the original 90-mer DNA (1.9 mG_0). The smaller conductivity of AT-rich DNAs as compared with that of GC-rich counterparts is commonly observed[5,6]. The prominent effect of the base sequence on the junction conductance proves that the electron transport as measured in the present study involves the orbitals of the DNA perpendicularly trapped in the electrode gap.

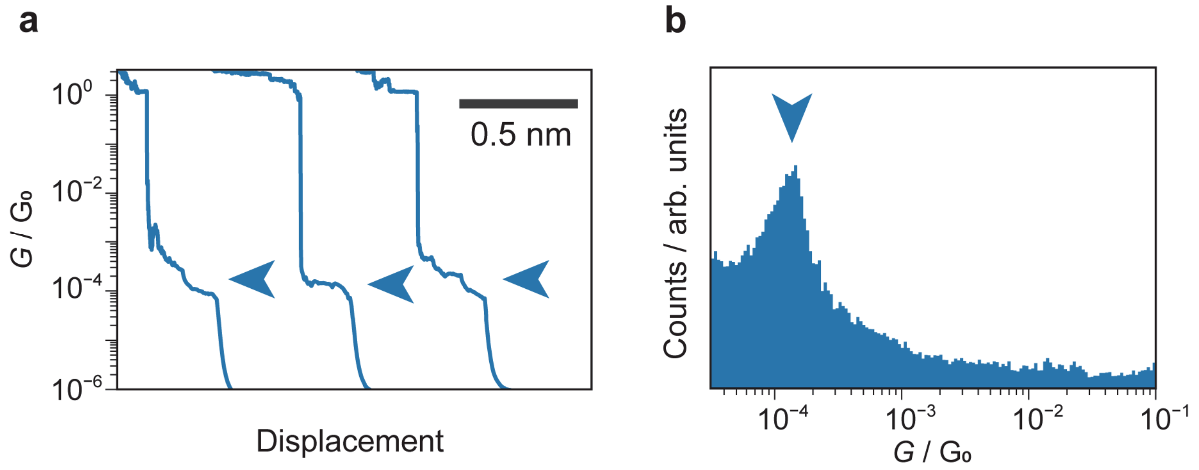

Supplementary Fig. 2. Typical conductance traces (**a**) and histogram (**b**) for "mutated" 90-mer DNA. Sequence, 5'-GCG CAA TGA AAG CCC GTG CCG TTA TCA GGC CGG ATT AGG TTA GAA TCG TGG AGC CAT TTT TTA AAA ATT TTT AAA AAT TTT TAA AAA–C_3SH-3', and its complement with C_3SH linker at 5' terminal. **b** Histogram was constructed from 1677 traces. Tip velocity, 10 nm/s; bias voltage, 20 mV.

Supplementary Note 3: DNA length dependence of conductance of zipper junction.

We measured the conductance of zipper junctions composed of 30-, 50-, and 70-mer DNA, in addition to the 10- and 90-mer DNA reported in the main text, by the STM-BJ technique to investigate the effect of DNA length on electron transport properties. For all of the investigated DNAs, a single peak appeared in the conductance histograms (Supplementary Fig. 3a), indicating the successful formation of zipper junctions (see Fig. 1a in the main text). Importantly, the conductance value increased as the DNA length increased (Supplementary Fig. 3b). The observed length dependence is in stark contrast with that of conventional single-molecule junctions of DNA. Most of the DNA junctions bear two linker groups, such as –SH, at the opposite terminals of the DNA. Electron transport steeply attenuates with increased DNA length in these cases, since the electrons travel through the whole duplex. However, in the present work, the linker groups were introduced at the same end of the DNA, and no such attenuation of electron transport happened. The increased

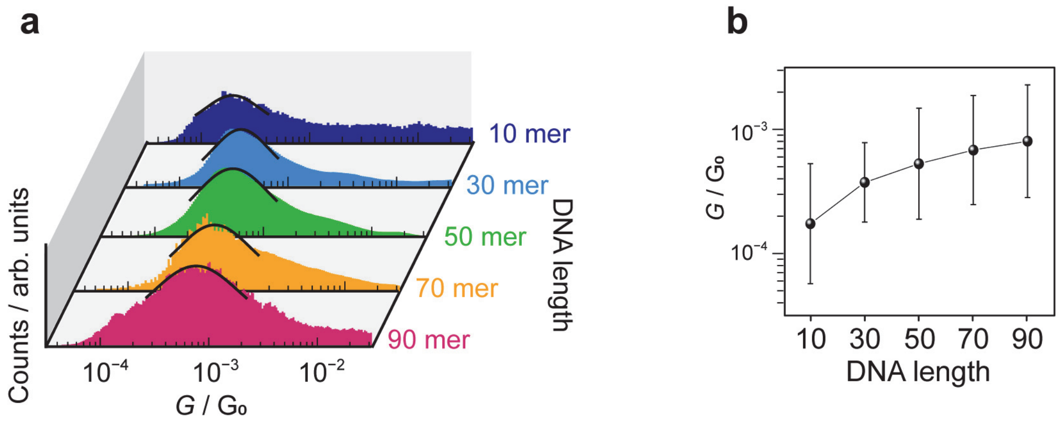

Supplementary Fig. 3. a Conductance histograms of DNA zipper junctions of 10-, 30-, 50-, 70-, and 90-mer DNAs, constructed from 2635, 1499, 999, 1999, and 2219 traces, respectively. Black lines show fitted Gaussian functions. **b** Length dependence of junction conductance. Black circles show mean conductance values of the Gaussian distribution, and error bars represent standard deviation of the distribution.

conductance can be explained based on transition voltage spectroscopy (Fig. 2d in the main text) and theoretical calculations (Supplementary Note 5).

Supplementary Note 4: Conductance of molecular junction as estimated by *I–V* curves.

To confirm the formation of the 90-mer DNA zipper junction in the *I–V* measurements as presented in Fig. 2a–c, we compared the conductance derived from the *I–V* properties and the conductance of the junction found in the STM-BJ experiments (Fig. 1). First, the *I–V* curves were clustered into two distributions using the fuzzy *c*-means algorithm[7,8], since two separate distributions were found in the V_{trans} histogram (Fig. 2). The successful classification is evident from the distinct difference between the *I–V* properties of the resultant sub-clusters (Supplementary Fig. 4a and b). The transition voltage analysis indicated that the high-conductance state corresponds to the DNA zipper junction, and the low-conductance state is ascribed to the tip-substrate gap without the molecular bridge. The conductance of each *I–V* curve in the sub-clusters was calculated, and the statistically most probable conductance values, G_{IV}, were found to be 3.1 and 4.0 mG_0 for the low- and high-conductance states, respectively, based on the histogram analysis (Supplementary Fig. 4c). In comparing these conductance values with corresponding values obtained by the STM-BJ study, care must be taken due to differences in the experimental conditions. Specifically, the tip-substrate distance was much smaller for the *I–V* measurements than for the STM-BJ measurements. This is because the present protocol to acquire the *I–V* curves is based on the so-called *I–t* measurements[9], in which the STM tip

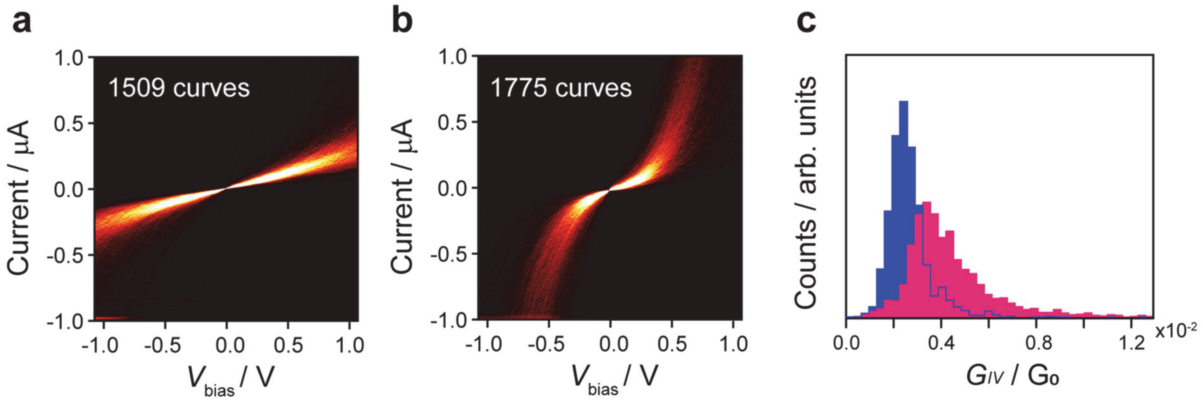

Supplementary Fig. 4. **a** and **b** Two-dimensional histograms of *I–V* curves of low- and high-conductance states, respectively. **c** G_{IV} histograms of the low- (blue bars and line) and high- (magenta bars) conductance states. The conductance was calculated as the slope of each *I–V* curve in low-bias region, from −100 to 100 mV.

is brought in close proximity to the sample surface to facilitate the spontaneous formation of the molecular junction[10]. Direct tunnelling between the metal tip and substrate is non-negligible under this condition, as evidenced by the relatively large conductance for the low-conductance state. We thus eliminated the contribution of direct tunnelling by considering the difference between the conductance of the high- and low-conducting states. The conductance of the molecular junction is consequently estimated to be 0.9 mG_0. This value is consistent with the conductance as determined by the STM-BJ experiments, which supports the assignment of the high-conductance state of the *I*–*V* property that arises due to the formation of the DNA zipper junction.

Supplementary Note 5: STM-BJ measurements using unmodified tip and DNA-modified substrate.

STM-BJ measurements were conducted using the unmodified tip and substrate modified with 90-mer single-stranded DNA (ssDNA). The sequence of the ssDNA was the same as that used in the STM-BJ experiments in the main text (Fig. 1). Supplementary Fig. 5a and 5b show the conductance traces and histogram, respectively. Clear plateaus and prominent peaks were found in the traces and histogram, respectively, at integer multiples of 1 G_0, which originated from Au point contacts between the tip and substrate. No reproducible plateaus were observed in the conductance traces below 1 G_0 unlike the case where both the tip and substrate were modified with complementary DNAs (Fig. 1). Consequently, no peaks appeared in the conductance histogram below 1 G_0, though the traces were measured with a wide conductance range by a logarithmic preamplifier. The slight increase in the histogram counts starting from approximately $10^{-2.5}$ G_0 were also found with the unmodified tip and unmodified substrate (black line in Supplementary Fig. 5b) and, thus, were

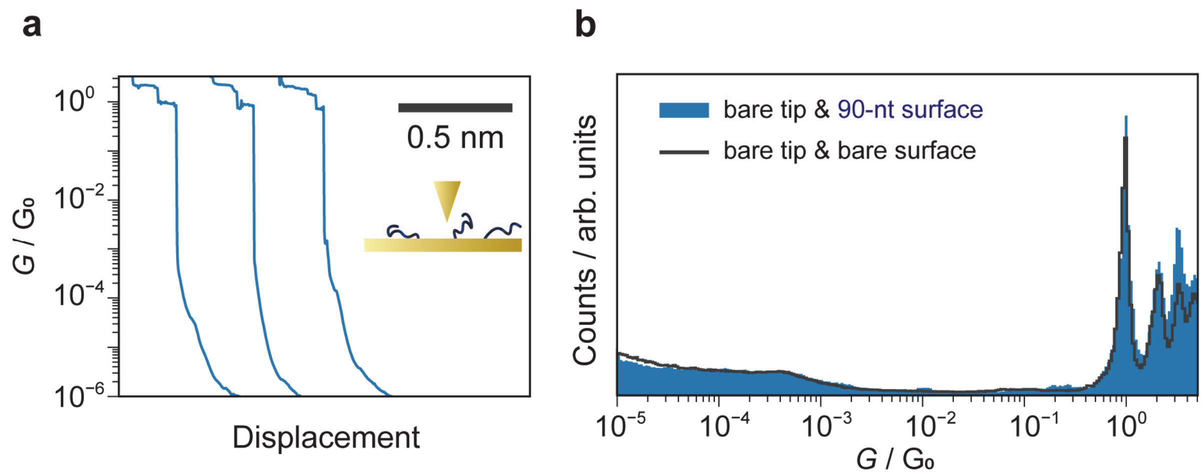

Supplementary Fig. 5. Typical conductance traces (**a**) and histogram (**b**) obtained with the measurements using the unmodified tip and ssDNA-modified substrate. **b** Histogram obtained with the unmodified tip and unmodified substrate was also shown by black solid line for comparison. The histogram counts were normalized by the values at 1 G_0. Histograms were constructed from 1653 and 2754 traces for the ssDNA-modified and unmodified substrate, respectively. Tip velocity, 10 nm/s; bias voltage, 20 mV.

attributed to background current noises. These results demonstrate that the ssDNA does not cause the formation of a single-molecule junction having detectable conductance values, most probably due to the significantly decreased conductance of ssDNA as compared to that of dsDNA.

Supplementary Note 6: Theoretical calculation of electronic structure of DNA.

The electronic structure of double-stranded DNA was theoretically investigated by means of density functional theory (DFT). The initial structures for the DFT calculations were prepared by molecular dynamics (MD) simulations using the AMBER package (version 16)[9]. We prepared 10-, 15-, 20-, 25-, and 30-mer DNAs with the same sequences as in our conductance experiments. The BSC1 force field[10] was used to describe DNA force field parameters, and Na^+ ions were placed at a position equally spaced from the two oxygen atoms of the phosphate group of the DNA backbone[11]. All MD simulations were performed with the implicit solvent based on the generalized Born model[12]. After the initial minimization, the equilibration processes were executed for 10 ns. The structures with the

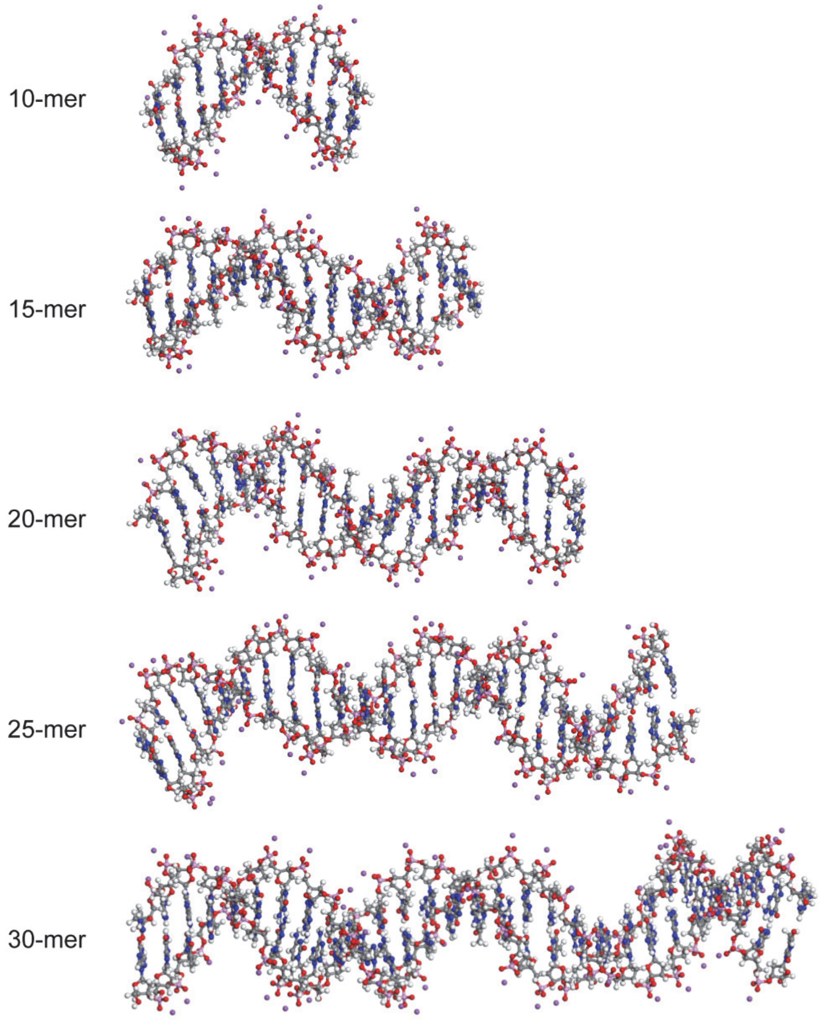

Supplementary Fig. 6. MD Snapshots of DNA structures with minimum total energies.

minimum total energies were extracted for the DFT calculation (Supplementary Fig. 6). The DFT calculation was performed with a double-numeric polarized basis set as the electronic wave function with a real-space cutoff of 0.5 nm using Dmol^3 code. The generalized gradient approximation (GGA) was used in the scheme of Perdew-Burke-Ernzerhof (PBE) to describe the exchange-correlation (XC) functional[13]. The solvation effects were considered based on the conductor-like Screening model (COSMO)[14,15], and a dielectric constant of 78.54 was used for solvation by water.

Supplementary Fig. 7 shows the energy-level diagram of the molecular orbitals (MOs) of dsDNA. It can be clearly seen that energy levels of the highest occupied MO (HOMO, E_{HOMO}) and the lowest unoccupied MO (LUMO, E_{LUMO}) increase and decrease, respectively, with the increase of the DNA length. The E_{HOMO} (or E_{LUMO}) of 30-mer DNA was increased by 0.079 eV (or decreased by 0.226 eV) compared to 10-mer DNA. Within the resonant level model, the electronic conductance becomes large, as the frontier orbital responsible for electron transport (either HOMO or LUMO in general) is energetically close to the Fermi level (E_F) of the electrodes[16]. The increase in the single-molecule conductance of the zipper junction with the longer DNA observed in the experiments (Fig. 1b in the main text and Supplementary Fig. 3b) is thus attributed to the increased E_{HOMO} and decreased E_{LUMO}, given that E_F lies in the HOMO–LUMO gaps. These results are consistent with

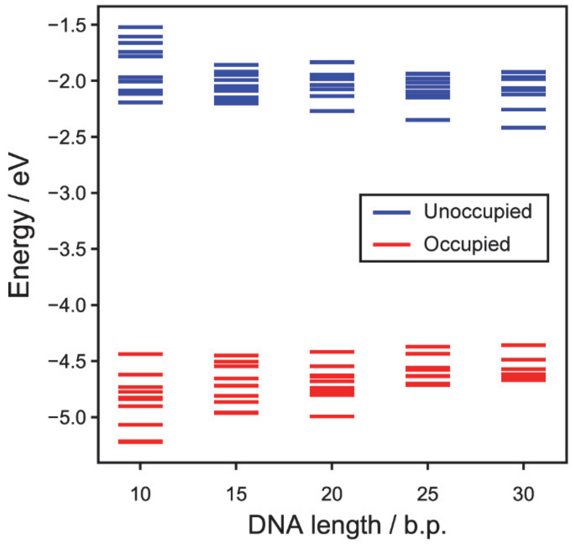

Supplementary Fig. 7. Energy diagram of 20 molecular orbitals around HOMO and LUMO of DNA with different length.

previous reports in which DNA bases with higher HOMO levels exhibit higher electrical conductance[17].

Supplementary Note 7: Joint-probability analysis of consecutive *G*–*z* traces.

Joint-probability analysis, adapted from the analysis of single-molecule fluorescence measurements,[18-20] was performed to evaluate temporal correlation in the formation of the molecular junction of the DNA zippers. First, the dwell length was estimated for each of the consecutive *G*–*z* traces. The resulting lengths were used to construct the 2D joint probability $g(l_1, l_2)$ to observe the dwell length l_2 after the dwell length l_1 in adjacent traces (Supplementary Fig. 8a and d for the 90-mer and 10-mer DNA zipper, respectively). The 2D joint probability $h(l_1, l_2)$ distributions were also prepared for two conductance traces separated by 50 measurements (Supplementary Fig. 8b and e for the 90-mer and 10-mer DNA zipper, respectively). In $h(l_1, l_2)$, the two dwell lengths, l_1 and l_2, almost lose correlation in-between because of the large separation. Supplementary Fig. 8c and f presents difference distributions, $g(l_1, l_2) - h(l_1, l_2)$, for 90-mer and 10-mer DNA, respectively. Importantly, Supplementary Fig. 8c exhibited large positive diagonal counts, indicating the presence of correlation between l_1 and l_2, in the dwell lengths around 0.1 nm. This length compares well with the average plateau length observed for the single-molecule junction of the 90-mer DNA zipper (Supplementary Fig. 1c). The distinct feature in the difference histogram demonstrates the high

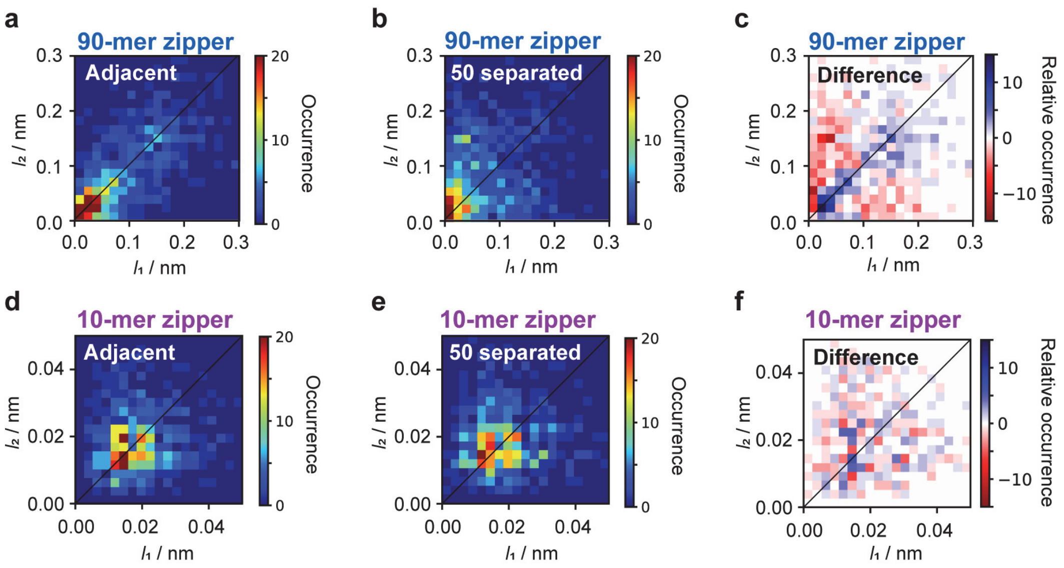

Supplementary Fig. 8. Joint-probability distributions of (**a** and **d**) $g(l_1, l_2)$ and (**b** and **e**) $h(l_1, l_2)$ of the consecutive *G*–*z* traces for 90-mer and 10-mer DNA, respectively. (**c** and **f**) Difference histograms, $g(l_1, l_2) - h(l_1, l_2)$ for 90-mer and 10-mer DNA, respectively.

likelihood to observe the long dwell length in the adjacent traces, i.e., the consecutive formation of the molecular junction for the 90-mer DNA zipper. In contrast, no distinct feature was found in the difference histogram for the 10-mer DNA zipper (Supplementary Fig. 8f), showing no correlation for the formation of the molecular junction of the DNA zipper in the consecutive *G–z* traces. The strong correlation between the adjacent dwell lengths in Supplementary Fig. 8c supports the repeated formation of the molecular junction of the 90-mer DNA zipper, as found in Fig. 3c.

Supplementary Note 8: AFM measurements of repeated formation of DNA zipper junction.

The self-restoration behavior of the DNA zipper junction was characterized by atomic force microscopy (AFM). An Au(111) surface modified with the double-stranded 90-mer DNA served as the sample surface. We first investigated the force–distance curves with an Au-coated cantilever (Supplementary Fig. 9a). The distinct rupture force demonstrated the successful formation of the DNA zipper junction between the tip and substrate, as discussed in the main text with a series of STM-based studies. A close inspection of the force–distance curves revealed the stepwise decrease in force by approximately 50 pN immediately before the complete rupture of the molecular junction (Supplementary Fig. 9b). Sawtooth-like force changes were also observed during the extension before the complete rupture (Supplementary Fig. 9c). We attributed these discrete changes to the

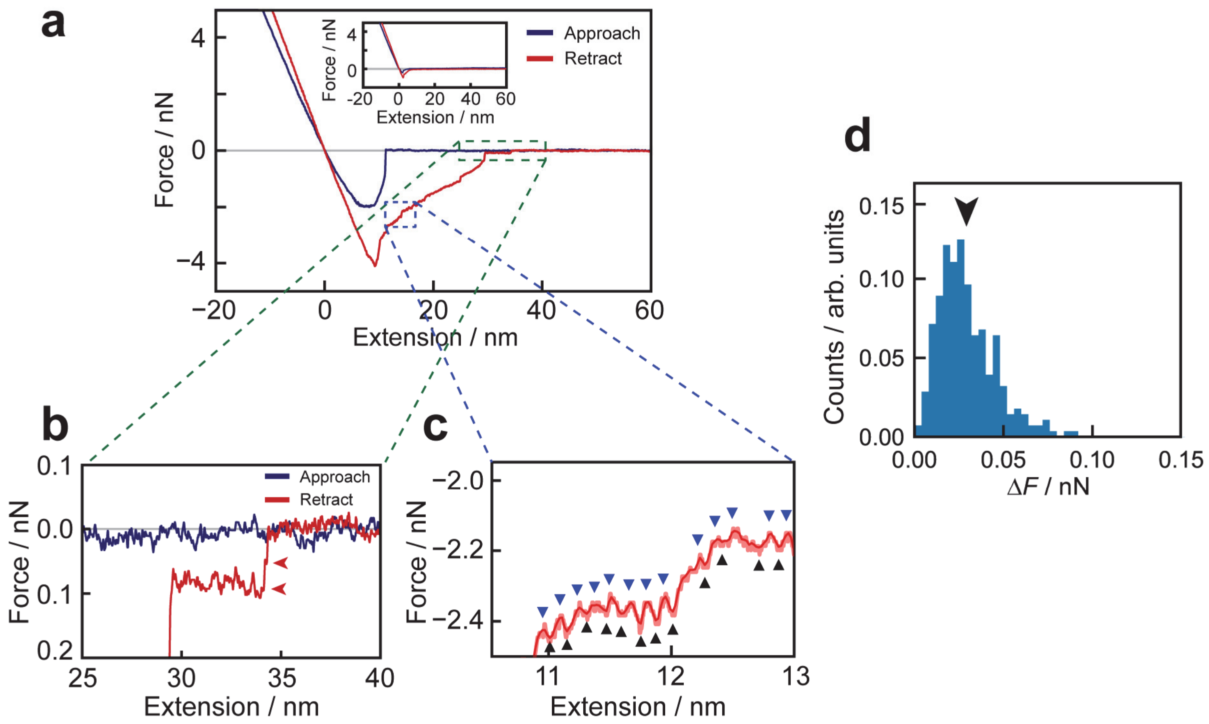

Supplementary Fig. 9. a Typical force–distance curve acquired with gold surface modified with 90-mer DNA zipper. Inset shows a force–distance curve for unmodified gold surface. **b, c** Enlarged view of force–distance in **a**. Arrowheads in **b** indicate the stepwise decrease in forces. In **c,** smoothed curve (dark red solid line) was overlaid on raw curve (light red solid line), and trianges represent sawtooth-like force changes. **d** Histogram of the force difference between adjacent peaks in **c**. The black arrowhead indicates the mean value.

force-induced melting of DNA duplexes in the junction. In fact, the statistically most probable force of 0.03 N found by the histogram analysis (Supplementary Fig. 9d) is consistent with the reported value for DNA melting[21-23]. The force curves measured in the present study are thus interpreted as a series of DNA melting from the molecular junction that initially contained hundreds of DNA duplexes.

Next, we addressed the repeatability of the formation of the zipper DNA: the force–distance curves were measured with an extension of 30 nm, which is insufficient to fully unzip the DNA duplex. The contour length of the 90-mer DNA is estimated to be 29 nm by assuming a rise distance of 0.33 nm/base-pair, but extensions longer than this contour length are necessary to mechanically melt the DNA duplex under the present linking configuration (see Fig. 1a in the main text). No complete rupture was observed in the resulting force–distance curves as expected (Supplementary Fig. 10a). Importantly, repeated approach–retract cycles resulted in force curves very similar to each other, indicating the restoration of the zipper DNA duplex (Supplementary Fig. 10b). The restoration behavior was quantitatively assessed by its probability defined by $1-(F_{re}-F_{app})/F_{re}$, where F_{re} and

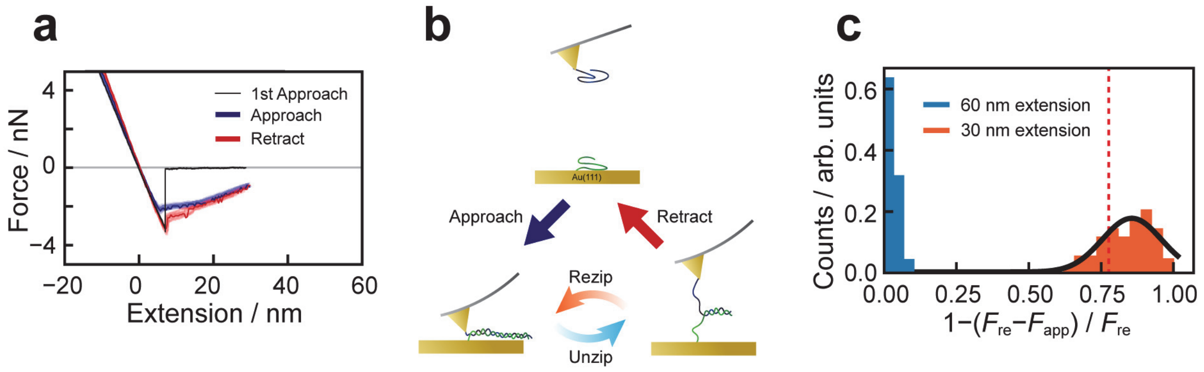

Supplementary Fig. 10. a Consecutive force–distance curves for Au(111) surface modified with 90-mer DNA zipper. 13 curves were shown. Cantilever extension of 30 nm was used. Averaged curves of approach and retraction processes were overlaid on raw curves. The black solid line shows the curve of the first approach process. **b** Plausible model for the formation and breakdown of the molecular junctions of DNA zipper. **c** Histograms of restoration probability for 30-nm (orange bars) and 60-nm (blue bars) extensions. The black solid line shows the fitted Gaussian function. The red dotted line indicates the restoration probability, estimated by STM experiments as shown in Fig. 3.

F_{app} are the force values at the displacement of 15 nm in the retraction and the subsequent approach processes, respectively. The histogram shows that 82% of the DNA zipper was restored during the re-approach after an extension of 30 nm (Supplementary Fig. 10c). A similar quantity was derived from the STM-BJ study. The restoration probability was directly measured by the occurrence of the successive formation of the molecular junction in the consecutive *G*–*z* measurements as presented in Fig. 3b and 3c in the main text. The probability was 77%, which is consistent with that found in the AFM study as argued above. In contrast, the restoring behavior was absent when an extension of 60 nm was applied in the approach–retract cycles. The results corroborate the repeated formation of the DNA zipper junction by self-restoration as observed in the STM-BJ experiments.

Supplementary Note 9: Displacement dependence of restoration capability of DNA zipper junction.

We investigated the relationship between the restoration capability of the DNA zipper junction and the tip displacement. The *G–z* measurements were performed with the STM tip modified with single strands of the DNA zipper. An Au(111) substrate covered with the complementary single strands served as the sample. The DNA tip was brought very close to the sample surface and pulled up to record the *G–z* traces. The displacements during the tip retraction were consecutively varied (10, 20, 40, 80, and 120 nm for the 90-mer DNA zipper; or 5, 10, 20, 40, and 80 nm for the 10 mer), and consequently, a single dataset contains five traces having different displacements. A representative example of the sequence of the *G–z* traces measured with the 90-mer DNA zipper is shown in Supplementary Fig. 11a. The presence or absence of the molecular junction of the DNA zipper was assessed based on the dwell length, i.e., the trace length between 1.4 mG_0 and 2.4 mG_0 and between 0.14 mG_0 and 0.16 mG_0 for the 90-mer and 10-mer zipper, respectively (see Supplementary Fig. 11b). The restoration capability was then quantitatively evaluated using a correlation coefficient of the dwell lengths taken from the two consecutive *G–z* traces. For example, the correlation coefficient for the 20-nm displacement, C_{20}, was calculated as

$$C_{20} = \frac{\sum_n \left(\Delta L_{20}(n) \Delta L_{40}(n)\right)}{\sqrt{\sum_n \left(\Delta L_{20}(n)\right)^2 \sum_n \left(\Delta L_{40}(n)\right)^2}} \quad 1$$

, where

$$\Delta L_x(n) = L_x(n) - \frac{\sum_n L_x(n)}{N} \quad 2$$

, and n, N, and $L_x(n)$ denote the index of the dataset, total number of the dataset, and the dwell length for the x nm displacement in the nth dataset, respectively. In the above example, the correlation coefficient for the 20-nm displacement involved the dwell length for the 40-nm displacement, because the restoration after the 20-nm displacement was reflected in the next *G–z* trace, i.e., the

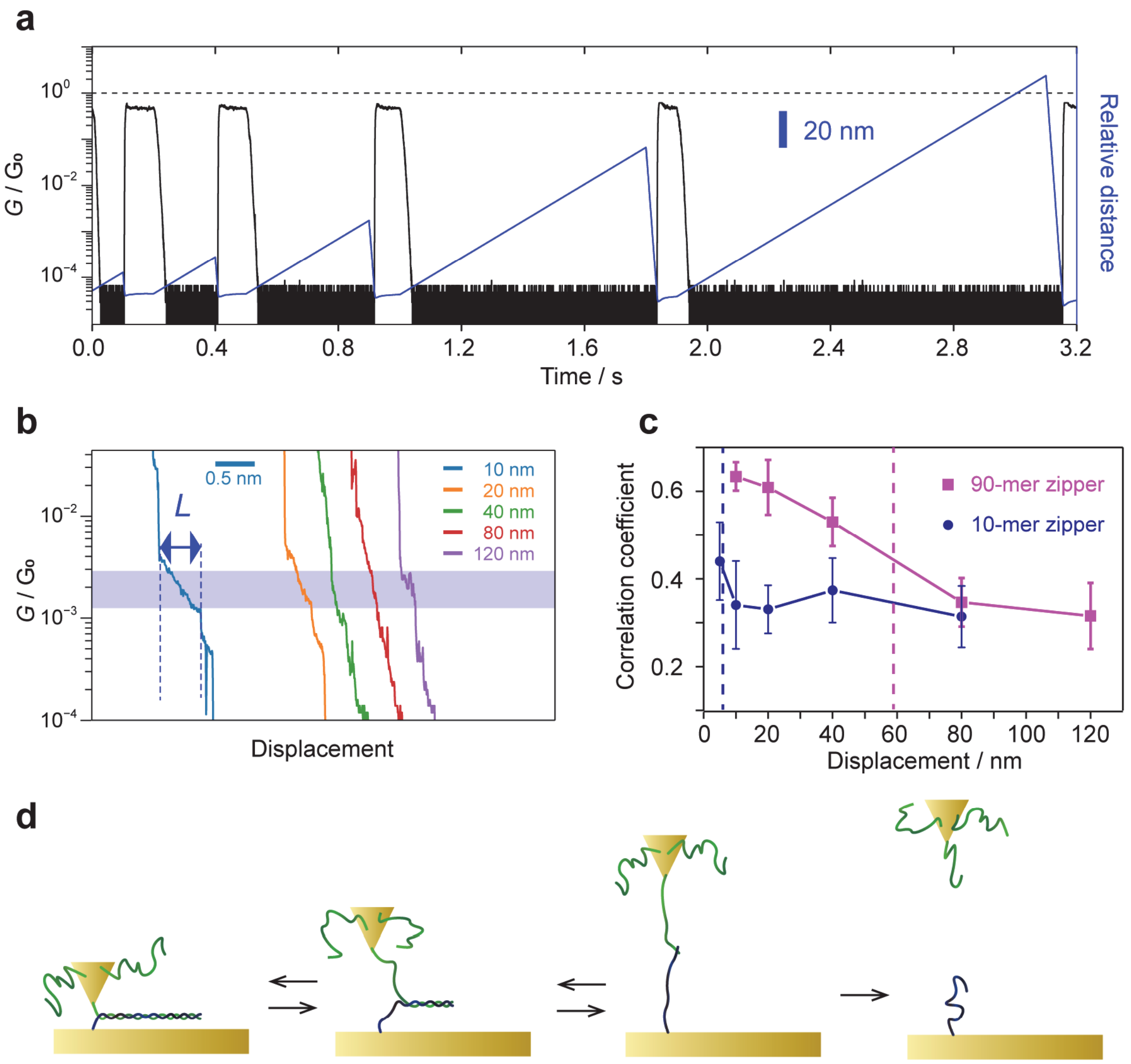

Supplementary Fig. 11. a Series of *G–z* traces (black) measured with 90-mer DNA zipper. Displacement of STM tip was also shown as blue solid line. Setpoint-current, 1000 nA; bias voltage, 20 mV. **b** *G–z* traces measured with different displacement extracted from dataset as in **a**. Purple-colored area indicates conductance range to determine dwell length. **c** Correlation coefficients of 10-mer (blue solid line) and 90-mer (pink solid line) DNA zipper. Dotted lines show maximum tolerable displacement for DNA zipper junctions. Four and eight datasets containing 200 sets of conductance traces were analyzed for 10-mer and 90-mer DNA zipper, respectively. Error bars represent standard error. **d** Schematic of structural evolution of DNA zipper junction during tip displacement.

trace for the 40-nm displacement. The correlation coefficients for the other displacements were similarly defined and calculated. In the case of the displacement of 120 nm for the 90-mer DNA

zipper (or 80 nm for 10-mer), whose traces were located at the end of each dataset, the dwell length of the first *G–z* trace (10-nm displacement for 90-mer, or 5-nm displacement for 10-mer) in the next dataset was used, along with the dwell length of the 120-nm (or 80-nm) displacement, for the calculation of the correlation coefficient. Supplementary Fig. 11c shows the resulting correlation coefficients of the 10-mer and 90-mer DNA zippers. For the 90-mer zipper, we found high correlation coefficients for shorter displacements and a gradual decrease for longer displacements. In contrast, in the case of the 10-mer zipper, the correlation coefficient was high only for the shortest displacement and decreased rapidly. The high correlation at the shorter displacements means that the DNA zipper junctions were regenerated during the two consecutive *G–z* measurements, demonstrating the restoration capability. It is noteworthy that the restoration behavior was observed even for the junction with 10-mer DNA, though at the short displacement. We thus anticipate that dsDNAs stable at room temperature can induce the restoration effect based on the present strategy.

The mechanism of the junction restoration can be inferred based on the observed displacement dependence. The maximum displacements for the DNA zipper junction without completely breaking the double strand can be estimated to be approximately twice the length of the DNA under the present junction structure (Supplementary Fig. 11d), i.e., 5.9 and 59 nm for the 10-mer and 90-mer DNA zippers, respectively. It is apparent from Supplementary Fig. 11c that the correlation was lost, and the coefficients decreased to the common background value of approximately 0.3 when the displacement exceeded the maximum tolerable distances. The gradual decrease in the correlation for the 90-mer DNA zipper junction until complete double-strand dissociation clearly indicates participation of the partially hybridized structures of the DNA zipper in the restoration behavior. In the *G–z* measurements with moderate displacements, the DNA zipper junction remained partially hybridized during the tip retraction. This structure facilitates the regeneration of the complete junction by re-hybridization[24-26]. The non-zero background of the correlation coefficient after the breakdown of the zipper junction is most probably attributed to the

use of the dwell length for their calculation, because the dwell length remained at a finite value even in the absence of the junction, which was due to direct electron tunnelling between the tip and substrate.

Supplementary Table 1: DNA sequences used in the experiments.

Name	Sequence
10-mer zipper	5′-GAC GGC CAC C-3′
30-mer zipper	5′-TCC ACA TCC GCT TGT GGT TTG ACG GCC ACC-3′
50-mer zipper	5′-GGT TAG AAT CGT GGA GCC ATT CCA CAT CCG CTT GTG GTT TGA CGG CCA CC-3′
70-mer zipper	5′-GCC GTT ATC AGG CCG GAT TAG GTT AGA ATC GTG GAG CCA TTC CAC ATC CGC TTG TGG TTT GAC GGC CAC C-3′
90-mer zipper	5′-GCG CCA TGA AAG AAG CCC GTG CCG TTA TCA GGC CGG ATT AGG TTA GAA TCG TGG AGC CAT TCC ACA TCC GCT TGT GGT TTG ACG GCC ACC-3′

Supplementary Note 10: Time-of-flight mass spectrum of synthesized DNA.

The DNA duplex was tethered to the electrodes (the STM tip and substrate) at the same end to create the zipper junction in the present work (Fig. 1a in the main text). To do so, the thiol-containing linker, that is, 1,3-propanethiol [-$(CH_2)_3SH$], was introduced at the hydroxy group of the 3′ end of one of the strand. The same linker was also introduced at the phosphate group of the 5′ end of another strand. These DNAs, synthesized according to the solid-phase synthesis using the phosphoramidite method and purified by high-performance liquid chromatography, were purchased from Tsukuba Oligo Service (Ibaraki, Japan). The purified products were characterized by time-of-flight mass spectrometry (TOF MS). Supplementary Fig. 12 shows TOF-MS spectra for the 90-mer strands. The measured mass of the molecular ion was consistent with the expected one, showing the successful introduction of the linker.

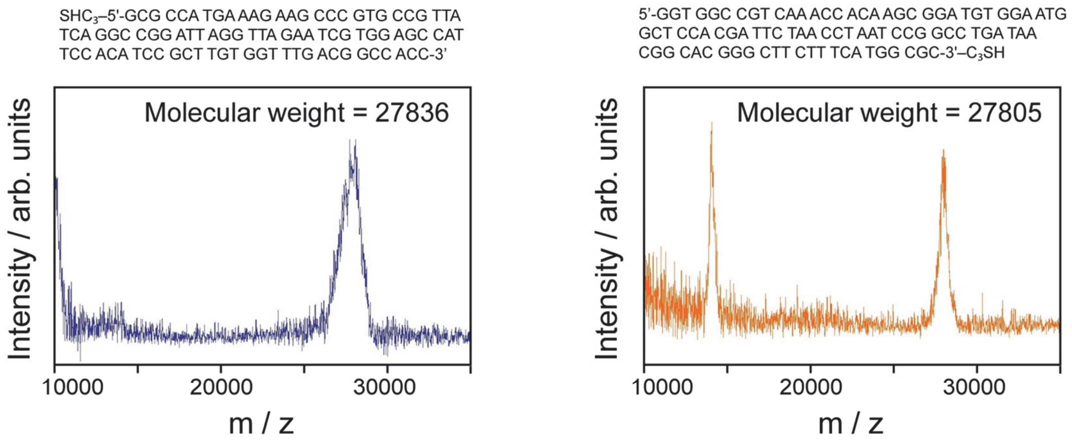

Supplementary Fig. 12. Time-of-flight mass spectrum of single-stranded DNAs used for creation of zipper junctions. Base sequences and molecular weights are also shown. –C_3SH stands for mercaptopropyl group (–$(CH_2)_3SH$).

Supplementary References

1. Kiguchi M. & Kaneko S. Single molecule bridging between metal electrodes. *Phys. Chem. Chem. Phys.* **15**, 2253-2267 (2013).

2. Bruot C., Xiang L., Palma J. L. & Tao N. Effect of Mechanical Stretching on DNA Conductance. *Acs Nano* **9**, 88-94 (2014).

3. Hatch K., Danilowicz C., Coljee V. & Prentiss M. Demonstration that the shear force required to separate short double-stranded DNA does not increase significantly with sequence length for sequences longer than 25 base pairs. *Phys. Rev. E* **78**, 011920 (2008).

4. Nath S. et al. Statistical mechanics of DNA rupture: Theory and simulations. *J. Chem. Phys.* **139**, 165101 (2013).

5. Hihath J., Xu B., Zhang P. & Tao N. Study of single-nucleotide polymorphisms by means of electrical conductance measurements. *Proc. Natl. Acad. Sci. USA* **102**, 16979-16983 (2005).

6. Chang S. et al. Tunnelling readout of hydrogen-bonding-based recognition. *Nat. Nanotechnol.* **4**, 297-301 (2009).

7. Lemmer M., Inkpen M. S., Kornysheva K., Long N. J. & Albrecht T. Unsupervised vector-based classification of single-molecule charge transport data. *Nat. Commun.* **7**, 12922 (2016).

8. Yang M. S. A survey of fuzzy clustering. *Math. Comput. Model.* **18**, 1-16 (1993).

9. Harashima T., Kojima C., Fujii S., Kiguchi M. & Nishino T. Single-molecule conductance of DNA gated and ungated by DNA-binding molecules. *Chem. Commun.* **53**, 10378-10381 (2017).

10. Haiss W. et al. Measurement of single molecule conductivity using the spontaneous formation of molecular wires. *Phys. Chem. Chem. Phys.* **6**, 4330-4337 (2004).

11. Anan R. et al. Change in binding states between catabolite activating protein and DNA induced by ligand-binding: molecular dynamics and ab initio fragment molecular orbital calculations. *J. Mol. Model.* **25**, 192 (2019).

12. Tsui V. & Case D. A. Theory and applications of the generalized Born solvation model in

macromolecular simulations. *Biopolymers* **56**, 275-291 (2000).

13. Perdew J. P., Burke K. & Ernzerhof M. Generalized Gradient Approximation Made Simple. *Phys. Rev. Lett.* **77**, 3865-3868 (1996).

14. Delley B. The conductor-like screening model for polymers and surfaces. *Mol. Simul.* **32**, 117-123 (2006).

15. Klamt A. & Schüürmann G. COSMO: a new approach to dielectric screening in solvents with explicit expressions for the screening energy and its gradient. *J. Chem. Soc., Perkin Trans. 2*, 799-805 (1993).

16. Evers F., Korytár R., Tewari S. & van Ruitenbeek J. M. Advances and challenges in single-molecule electron transport. *Rev. Mod. Phys.* **92**, 035001 (2020).

17. Albrecht T. Electrochemical tunnelling sensors and their potential applications. *Nat. Commun.* **3**, 829 (2012).

18. English B. P. et al. Ever-fluctuating single enzyme molecules: Michaelis-Menten equation revisited. *Nat. Chem. Biol.* **2**, 87-94 (2006).

19. Lippitz M., Kulzer F. & Orrit M. Statistical evaluation of single nano-object fluorescence. *Chemphyschem* **6**, 770-789 (2005).

20. Lu H. P. Single-Molecule Enzymatic Dynamics. *Science* **282**, 1877-1882 (1998).

21. Gaub H. E., Rief M. & Clausen-Schaumann H. Sequence-dependent mechanics of single DNA molecules. *Nat. Struct. Biol.* **6**, 346-349 (1999).

22. Krautbauer R., Rief M. & Gaub H. E. Unzipping DNA Oligomers. *Nano Lett.* **3**, 493-496 (2003).

23. Bockelmann U., Thomen P., Essevaz-Roulet B., Viasnoff V. & Heslot F. Unzipping DNA with Optical Tweezers: High Sequence Sensitivity and Force Flips. *Biophys. J.* **82**, 1537-1553 (2002).

24. Ansari A., Kuznetsov S. V. & Shen Y. Configurational diffusion down a folding funnel

describes the dynamics of DNA hairpins. *Proc. Natl. Acad. Sci. USA* **98**, 7771-7776 (2001).

25. Wetmur J. G. & Davidson N. Kinetics of renaturation of DNA. *J. Mol. Biol.* **31**, 349-370 (1968).

26. Yin Y. & Zhao X. S. Kinetics and Dynamics of DNA Hybridization. *Acc. Chem. Res.* **44**, 1172-1181 (2011).
