# Supplementary material for: Single-molecule junction spontaneously restored by DNA zipper
Source: Nat Commun. 2021 Oct 1;12:5762. doi: 10.1038/s41467-021-25943-3 (PMC8486845; doi:10.1038/s41467-021-25943-3)
Supplement: Supplementary file 3 — Description of Additional Supplementary Files [file 41467_2021_25943_MOESM3_ESM.pdf]

Description of Additional Supplementary Files

File Name: Supplementary Movie 1
Description: Molecular dynamics simulation for reannealing process of DNA zipper under external forces.

File Name: Supplementary Movie 2
Description: Molecular dynamics simulation for reannealing process of DNA zipper without external forces.
